# Physiotherapy Intervention on Premature Infants—A Pilot Study

**DOI:** 10.3390/medicina60010138

**Published:** 2024-01-11

**Authors:** Daniela Parau, Anamaria Butila Todoran, Rodica Balasa

**Affiliations:** 1 Doctoral School, ‘George Emil Palade’ University of Medicine, Pharmacy, Science, and Technology of Targu Mures, 540142 Targu Mures, Romania; 2 Department of Genetics, ‘George Emil Palade’ University of Medicine, Pharmacy, Science, and Technology of Targu Mures, 540142 Targu Mures, Romania; 3 Department of Neurology, ‘George Emil Palade’ University of Medicine, Pharmacy, Science, and Technology of Targu Mures, 540136 Targu Mures, Romania; rodica.balasa@umfst.ro

**Keywords:** premature, motor development, physiotherapy, Bobath, infants

## Abstract

*Background and Objectives*: Considering the fact that prematurity echoes in terms of motor development even up to the age of adolescence, through the presence of deficiencies, the importance of starting kinetotherapeutic treatment as soon as possible is highlighted, even in the absence of brain damage or obvious motor delays. Therefore, the objectives of this study are to analyze the factors that influence the level of motor development of premature babies up to 9 months and identify the motor development curve of premature babies according to the three stages of motor development: the position of symmetrical support on the elbows at 3 months, sitting with support at 6 months, and verticalization at 9 months. *Materials and Methods*: This prospective pilot study was conducted within a rehabilitation facility located in Targu Mureș, Romania, spanning a duration of 2 years from June 2021 to 2023. *Results*: The study involved a population of 78 children, all premature infants, selected from the patient pool of the rehabilitation facility, specifically chosen based on adherence to the predetermined inclusion and exclusion criteria outlined in the study protocol. Two physiotherapists specialized in child recovery were involved in the study, and one performed the assessments and the other applied the Bobath therapy. *Conclusions*: Early physiotherapy interventions can have a positive influence in terms of reducing differences in motor development between preterm and full-term infants. This study identified several factors that influence the motor development of premature infants. Among these, the most prominent biological factors were gestational age and birth weight.

## 1. Introduction

Considered a worldwide epidemic, preterm birth, defined by the World Health Organization (WHO) as birth before the gestational age of 37 weeks, has an incidence of 15 million births in one year [1]. 

The specialized literature [2] classifies premature birth according to the duration of gestation; thus, in practice, it is encountered as follows: extreme prematurity (gestational age < 28 weeks, approximately 5% of births), high prematurity (gestational age between 28 and 31 weeks, approximately 10%), moderate prematurity (gestational age between 32 and 33 weeks), approximately 85% [3], and late prematurity (gestational age between 34 and 36 weeks, their percentage being included in that mentioned for moderate prematurity). Despite the reported associations between preterm birth and a wide range of socio-demographic, medical, obstetric, fetal, and environmental factors, approximately two-thirds of preterm births occur without an obvious risk factor [3]. The most relevant factors [4] that can induce premature birth are as follows: preeclampsia or eclampsia, intrauterine growth restriction, premature births prior to infection or inflammation, vascular diseases and uterine overdistension, premature rupture of membranes, genetic predispositions [5], premature multiple pregnancies associated with assisted reproduction technologies, and in vitro fertilization [6].

Although medical practices and neonatal care have progressed in recent years, resulting in a higher survival rate for babies born prematurely, this situation constitutes a new challenge to the medical system in light of the fact that the survival rate of premature babies (especially large premature babies born before 32 weeks) is accompanied by an increase in the number of children with disabilities [7].

Annually, 5–8% [7] of survivors born very prematurely develop cerebral palsy (CP) [8], which is the leading cause of disability in childhood, leading to significant delays and/or disturbances in postural, manual, and locomotor development [8,9]. Even if they do not develop CP, very preterm infants remain at risk of developing motor problems, with 40% [10] showing developmental delays or disturbances, including later sensory-motor abnormalities that may affect gross motor development. Also, the risk of motor or cognitive impairment is two to three times higher in children born between 34 and 36 weeks than in children born at term [2]. Therefore, every premature baby must be carefully monitored. Recent cohort studies have focused on extremely preterm births. Awareness of the potential outcome and prognosis of all preterm infants is a crucial step for healthcare professionals caring for these infants [2].

Considering the fact that prematurity echoes in terms of motor development even up to the age of adolescence, through the presence of deficiencies, the importance of starting kinetotherapeutic treatment as soon as possible was highlighted, even in the absence of brain damage or obvious motor delays [11,12].

Therefore, the objectives of this study are as follows:

1. Analysis of the factors that influence the level of motor development of premature babies up to 9 months.
2. Identification of the motor development curve of premature babies according to the three stages of motor development: the position of symmetrical support on the elbows at 3 months, sitting with support at 6 months, verticalization at 9 months, and identifying the factors that influence motor development in premature infants.

In practice, several therapeutic approaches are used on premature infants, of which the Bobath method was chosen in the present study. The Bobath concept was developed by the spouses Karel and Bertha Bobath, a neurologist and a specialist physiotherapist, based on an analysis of the causes of cerebral palsy. The two concluded that the factors that lead to loss of control over the muscular system include sensory disturbances of various degrees, spasticity, disorder in the postural reflex mechanism, and a lack of selective movement modalities [13].

According to the authors’ perspective, the therapy mechanism is based on the fact that the state of the body’s muscles is reflected at every moment by the central nervous system [14]. The state of muscle stretching and contraction generates the distribution of inhibitory and excitatory processes in the nervous system, causing a flow of excitation to the periphery. The reflex inhibitory postures have large joints as key points, so that muscle tone is reduced in the limbs and spasticity is eliminated. The Bobath method accompanies the child in all its developmental stages, including rolling, crawling, sitting up, walking on all fours, walking on the knees, standing upright, and walking independently, with the aim of achieving physiological mobility [15].

Bobath therapy is based on three principles [16]: inhibition of tonic reflex activity, stimulation of the body’s means of balance reaction, and fixing recovery objectives.

Inhibition of reflex tonic activity. This is achieved by identifying positions that inhibit reflexes for the patient, thus unblocking the channel of nerve impulse flows and opening new pathways for normal reflex activity. The reflex-inhibitory positions are antagonistic to the patient’s abnormal posture, and, according to the authors’ view, they are applied globally and not only in the case of the affected limb.

Setting recovery goals. This principle is based on the fact that setting recovery goals for infants must be based on ideal neuro-motor development stages. Therefore, the delay in neuro-motor development is identified by comparison with the normal developmental stages that infants go through, and the goals of recovery are established according to the developmental stages that the patient needs to follow.

By applying this therapy, it is possible to prevent the occurrence of further complications, contractures, and deformations [17,18].

Research question: infants born prematurely face neuromotor delays, and their early involvement in a physical therapy program can mitigate developmental differences compared to the child’s ideal developmental ontogeny.

## 2. Materials and Methods

This prospective pilot study was conducted within a rehabilitation facility located in Târgu Mureș, Romania, spanning a duration of 2 years from June 2021 to 2023. The study involved a population of 78 children selected from the patient pool of the rehabilitation facility, specifically chosen based on adherence to the predetermined inclusion and exclusion criteria outlined in the study protocol.

Two physiotherapists specialized in child recovery were involved in the study; one performed the assessments and the other applied the Bobath therapy. The study was carried out in accordance with the Declaration of Helsinki, and the study protocol was approved by the Scientific Ethics Committee of the University of Medicine, Pharmacy, Science and Technology “George Emil Palade” of Târgu Mureș, no. 962/03.06.2020. Written consent was obtained from the guardians of all patients before participation in the study.

Participants: The study group was represented by 78 infants aged between 0 and 9 months, all premature (gestational age between 34 and 36 weeks), with a weight between 1500 g and 2500 g and the presence of neuro-reflexo-motor delay. Of these, 36 infants aged between 0 and 3 months constituted the group that benefited from therapy, and 42 infants aged 9 months who were not subjected to therapy represented the control group.

Before inclusion in the study, infants were assessed by a neurologist to identify whether they exhibited any evident neurological conditions. Additionally, their reflexes and muscle tone were evaluated.

Inclusion criteria: The therapy group included infants aged between 0 and 3 months, born at a gestational age of 34–36 weeks, and weighing between 1500 g and 2500 g, with an Apgar score between 8 and 10 in the first minute. The control group included infants aged 9 months, born at a gestational age of 34–36 weeks, and weighing between 1500 g and 2500 g, with an Apgar score between 8 and 10 in the first minute. The written consent of the relatives was obtained for all these children.

Exclusion criteria: obvious neurological conditions, gestational age at birth less than 34 weeks, weight less than 1500 g, and genetic syndromes.

Initially, 57 infants were included in the therapy group, but during the course of the study, they were excluded: three infants who were subsequently diagnosed with congenital hip dysplasia, requiring discontinuation of therapy due to immobilization in the orthotic system; six infants suffered digestive disturbances requiring hospitalization; three infants developed cardiac complications and exercise was contraindicated; four infants developed respiratory complications (respiratory distress); and five others dropped out of therapy along the way.

The physiotherapist responsible for the clinical assessment (initial, intermediate, and final) observed and noted each participant in an evaluation form containing the age and weight at birth, gender, Apgar score, muscular tone, the initial motor development stage, and the motor evolution up to, and including, 9 months of age.

It was established according to the clinical picture manifested by the infants and compared with the one described in the grid of ideal motor development designed by Dr. Vojta V. Infants were positioned in supine and prone positions, and spontaneous whole-body motricity and movement sequences were analyzed. The evaluations focused on the following quantitative and qualitative aspects: assessing the degree of neuro-motor development, observing the reaction to reflexes in the dorsal and ventral decubitus, recording motor progress in achieving specific positions for each age stage, comparing the symmetry of movements, and analyzing the correctness of posture and movement control.

Inclusion in a stage of neuromotor development below the infant’s age was determined when the infant did not meet the motor criteria described by Vojta for that age range. Additionally, if the infant lacked adequate motor control and did not perform movements symmetrically or correctly, even if they executed the specific positions and movements for the 0–9 month age range, they were categorized as having a lower neuro-motor development level.

Since the time of the assessment overlapped with the onset of therapy, there were infants aged 0–2 months who were assessed four times, once at entry into therapy and subsequently at 3–6–9 months of age. The rest of the participants in the therapy group, who were included in the study at 3 months, were evaluated three times (at the age of the infants of 3–6–9 months). Premature infants in the control group were assessed only once at 9 months of age.

The analysis of the data obtained from the evaluations focused particularly on highlighting the neuromotor gap between the chronological age of infants and the age of neuromotor development.

Later, the information obtained from the evaluations was organized and structured with the help of databases using the Excel program.

Recovery program: To the infants in the therapy group, Bobath therapy was applied with a duration of 30 min/session and a frequency of three times/week, while on the rest of the days, parents were instructed in certain simple procedures for home-based self-therapy. The rehabilitation program was individually tailored for each premature infant based on their neuromotor developmental stage. For infants exhibiting pathological reflexes, inhibitory reflex positions were employed. These maneuvers were applied to infants aged between 0 and 9 months.

The session included exercises in various positions such as dorsal decubitus (DD), lateral decubitus (DL), ventral decubitus (DV), sitting, on all fours, kneeling, standing, and using assistive devices such as a therapy table, mattress, Bobath ball, inflatable cylinder, disc Sveltus balance, inflatable balance disc, trellis, and treadmill recovery.

Bobath therapy focused on improving automatic postural reactions, lifting and balancing reactions, and adaptive changes in muscle tone [19].

The exercises conducted for infants aged between 0 and 9 months were personalized according to the level and initial functional deficit of each patient and consisted of the following mobilizations: Positions that inhibit pathological reflexes are partially opposite to the infant’s abnormal position. First, the head and neck were adjusted, followed by the torso, shoulders, and hips, to redistribute muscle tone as close to normal as possible.

- With head adjustment, the tonic reflexes of the neck were activated to favor flexion or extension of the upper or lower limbs.
- In order to activate the asymmetrical neck reflexes, the head was positioned on the side of the affected limb to achieve relaxation of the flexor tone. Alternatively, mobilization was facilitated by turning the head in the other direction.
- To stimulate the tonic labyrinthine reflex, the infant was positioned in the supine position (DD), and through anterior flexion of the head and neck, with the upper limbs crossed on the ribcage, relaxation of the lower limbs was achieved.
- In the case of the opisthotonus position, to relax the extensor muscles of the neck and at the level of the trunk and limbs, the fetal position was adopted and performed with slight antero-posterior rocking movements.
- For an infant who showed a tendency to lean forward, the lifting was performed with maintenance in the axillary area to obtain the extension of the head and limbs in a reflex inhibitory position, thus favoring easy movement of the limbs. The same relaxation was obtained from the recumbent position ventral (DV) by lifting the infant’s head and supporting the abdomen.

The exercises designed to enhance postural control of the head, neck, stability of the scapulo-humeral girdle, and support on the upper limbs for infants aged 0–3 months involved prompting them to lift and move their heads in various angles and directions. These movements were facilitated through maneuvers inducing body imbalance, performed on the Bobath ball.

For infants aged between 3 and 6 months, exercises in the prone position were conducted to achieve a posture with support on the elbow and palms. These exercises involved direct and indirect lifts, flexion and extension of the arm, and dynamic sways of the body, both forward and backward, as well as lateral tilts to the left and right. Rolling exercises from the dorsal decubitus to the lateral decubitus were performed in both directions, with a gradual transition from the lateral decubitus to the ventral decubitus to facilitate rolling. To lift with support on the forearms and elbows, followed by lifting with support on the palms with the upper limb as extended as possible, undulatory movements were induced using devices such as an inclined plane and the Bobath ball. These movements were characterized by spiral rolling and rolling of the body and its segments.

For infants aged between 6 and 9 months, a set of exercise complexes that have been used to stimulate the body’s balancing reactions through a variety of means, eliciting and strengthening them through repetition, was implemented.

**Figure 1:**
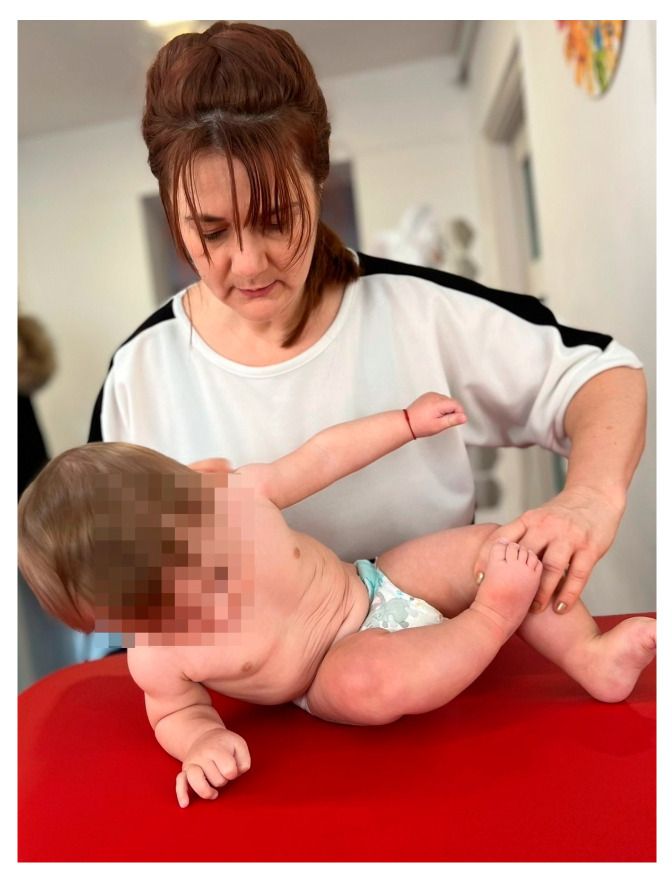
Sitting position.

**Figure 2:**
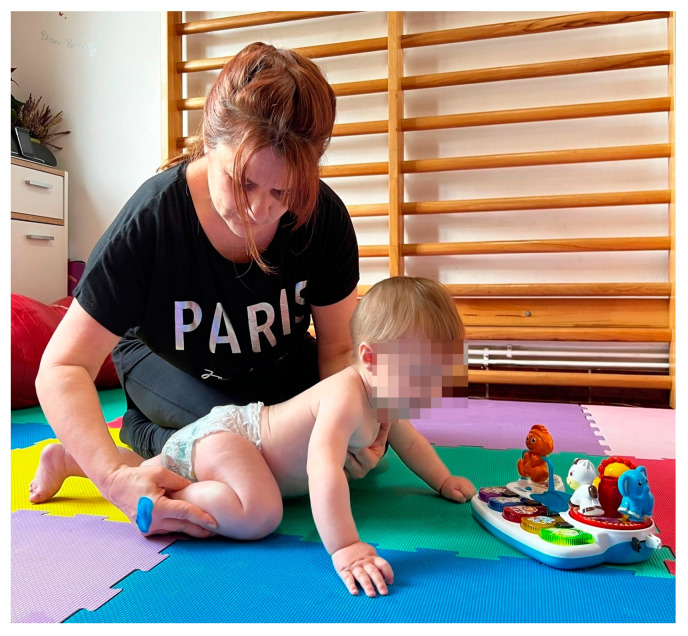
Quadruped positioning.

**Figure 3:**
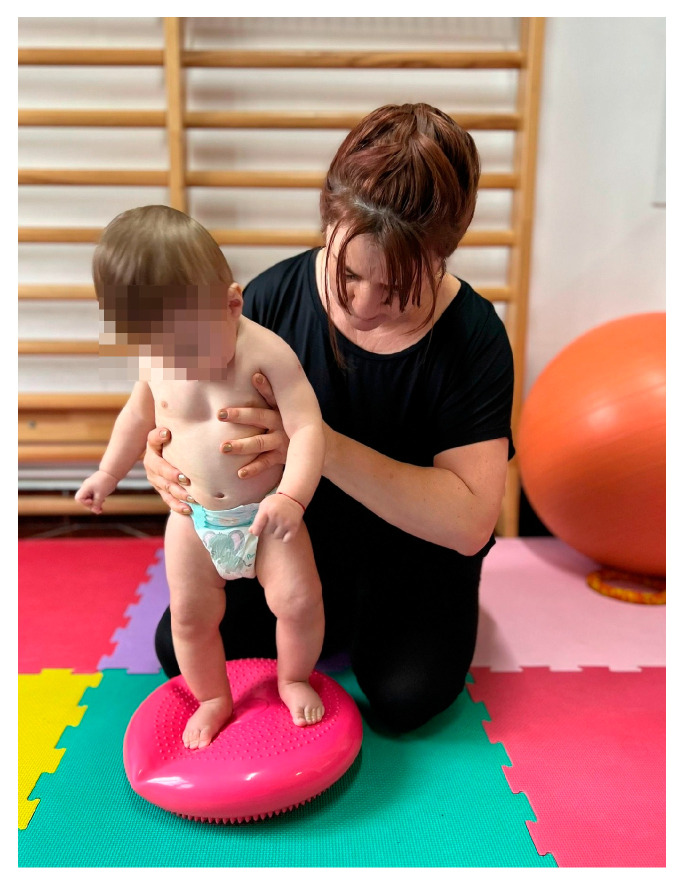
Balance stimulation.

- Small, brief pressures were applied to the infant’s shoulder while sitting, kneeling, or in a quadruped position, pushing it in different directions to teach it to respond by raising its arm in the direction of the push. This exercise was performed in two sets of five repetitions, with a one-minute break between sets.
- In order to stimulate the infant to sit up from the supine position, its return to the lateral decubitus was initiated, with support on the body, elbow, and then on the palm, while being maintained at the level of the lower limbs by the physiotherapist. This exercise was performed in two sets of five repetitions, with a one-minute break between sets (Figure 1).

- In order to transfer from the sitting position to the quadrupedal position, the infant was given a lateral movement either to the left or to the right, with progressive loading of the upper limbs. This movement can be performed on a horizontal surface or on a slope, tilted down. The exercise was performed in two sets of five repetitions, with a one-minute break between sets.
- From the ventral decubitus position, transitioning the infant into quadrupedal posture was achieved with support in the anterior thoracic region, facilitating loading on the upper limbs. Simultaneously, by gently elevating the pelvis through flexion of the hip joints, loading at the knee level was facilitated. This exercise was performed in sets of five repetitions, either on a mat or with the assistance of a Bobath ball (Figure 2).

- The initiation of verticalization was achieved through the “Servant Knight” position. The initial position involved support on both knees and on the upper limbs. The infant was lifted through one of the flexed lower limbs (triple flexion at the hip, knee, and ankle) and pushed to adopt a balanced and stable position, being supported by both supporting limbs. This exercise was performed in two sets of five repetitions for each lower limb, with a one-minute break between sets (position executed only at 9 months).
- In the standing position, an inflatable disc was used to stimulate balance. Because balance is an essential reaction for walking, the infant was slightly thrown off balance by anteroposterior and lateral thrusting movements, a position executed only at 9 months (Figure 3).

### Statistical Analysis

The software that was used for the statistical processing of the study data was IBM SPSS Statistics for Windows, Version 29.0. (30-day trial version) Armonk, NY, USA: IBM Body.

Nominal data were presented as absolute frequency and percentage, and continuous variables were expressed as mean and standard deviation.

Analysis of the association between categorical variables was performed using cross-tabulation and the χ^2^ (chi-square) test. If the results of the chi-square test were sufficiently altered that they could not be considered, Fisher’s exact test was used.

In view of the independent samples, the *t*-test was used to compare the means according to the numeric variables in the study. To compare three or more group means where the participants are the same in each group, we used the ANOVA test. After applying the ANOVA procedure, to indicate to what extent the means of the two-by-two groups differed, we applied the Bonferroni post hoc test. A statistical significance coefficient value of *p* < 0.05 was considered significant.

## 3. Results

The study group included subjects of both genders, with the female representatives being 38.9% and the male representatives being 61.1%. Almost half of the newborns (47.2%) were born after 34 weeks of gestation, 30.6% after 35 weeks, and 8 (22.2%) after 36 weeks of gestation. A total of 58.3% of preterm infants had an APGAR score of 8 at birth, 25% had an APGAR score of 9, and 16.7% achieved the maximum score. The mean age of the preterms included in the study at birth was 34.75 weeks. The average weight was 1896.94 g, while the APGAR score had an average of 8.58.

Half of the preterm infants had hypotonic muscle tone, while the other half had spastic muscle tone. At the initial assessment, 19.4% of the preterm infants were 1 month old, 30.6% were 2 months old, and 50% were already 3 months. At the initial assessment, the motor development of 52.8% of premature infants was at 0 months, 25% was at 1 month, 11.1% was at 2 months, and 11.1% was at 3 months. At the age of 3 months, 22.2% of prematures had motor development like that of a 0-month-old, 30.6% like that of a 1-month-old, 36.1% like that of a 2-month-old, and only 11.1% like that of a 3-year-old. At the 6-month assessment, 16.7% of preterm infants had motor development similar to that of a 3-month-old, 30.6% to that of a 4-month-old, and 52.8% to that of a 5-month-old. At the assessment performed at 9 months of age, 41.7% of preterm infants had motor development similar to that of a 7-month-old, 33.3% to that of an 8-month-old, and 25% to that of a 9-month-old (Table 1).

**Figure 4:**
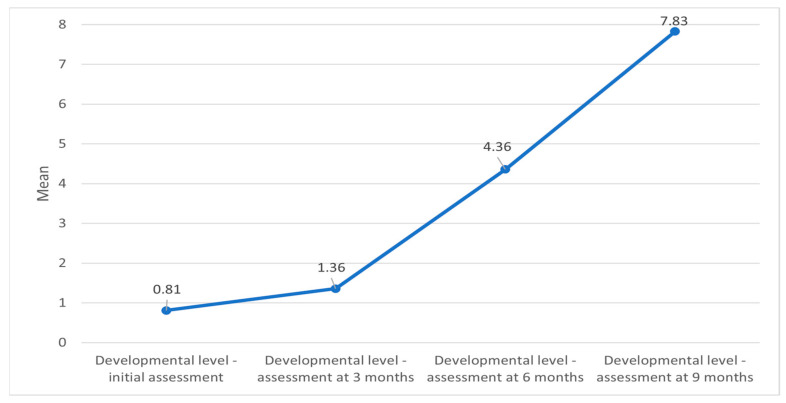
Motor development curve.

The data presented in Figure 4 include numerical values representing the average levels, which have been measured or assessed at various time intervals: initially, at 3 months, at 6 months, and at 9 months. The average levels of neuromotor development recorded in infants evolved from 0.81 at the initial assessment to 1.36 at the 3-month assessment, then to 4.36 at the 6-month assessment, and finally to 7.83 at the assessment conducted at 9 months (Figure 4). The results showed that the evolution of motor development was statistically significant (*p* < 0.001) in the sense of its improvement and approach to ideal motor development.

In Table 2, the level of motor development can be followed at the ages of 3–6–9 months depending on the gender of the premature babies.

The presence of a significant association between the two variables at the age of 3 months was found. More precisely, the level of motor development at 3 months is associated with the gender of the premature infant (*p* = 0.009, χ^2^ = 11.577, df = 3). Males with a very low level of development were in a higher proportion (36.4%) than females.

However, no significant association was found between the level of motor development at the age of 6 months and the gender of premature infants (*p* = 0.081, χ^2^ = 5.035, df = 8). Regardless of gender, the levels of motor development at 6 months are similar.

No significant association was found between the level of motor development at the age of 9 months and the gender of premature babies (*p* = 0.839, χ^2^ = 0.351, df = 2). The level of motor development at 9 months did not differ according to the gender of the child.

A lower level of motor development at 3 months was observed in a higher percentage of preterm infants born at 34 weeks, while preterm infants born at 36 weeks mostly had higher motor development (Table 3).

The result of the statistical analysis also shows statistically significant differences (χ^2^ = 22.210; df = 6; *p* = 0.001). Therefore, we can say that there is an association between the age at birth and the level of motor development.

While all preterm infants born at 36 weeks had a level of motor development at 6 months, equivalent to 5 months of age, high proportions of those born at 34 and 35 weeks, respectively, have low levels of motor development (Table 3). A significant association was also found between the age at birth and the level of motor development at the age of 6 months (χ^2^ = 9.654; df = 4; *p* = 0.047) (Table 3).

In Table 3, the share of premature babies born at 36 weeks who reached a developmental level equivalent to this age at 9 months was significantly higher (75%) than in the case of premature babies born at 34 weeks (17.6%), respectively, 35 weeks (0.0%). Differences were also confirmed by the result of the statistical analysis (χ^2^ = 28.792; df = 4; *p* < 0.001; Cramer’s V = 0.632).

A lower APGAR score at birth is accompanied by a lower level of motor development at the 3-month assessment (Table 4).

To test the association between the APGAR score and the level of motor development at 3 months, the chi-square test was used. Since *p* < 0.05, the presence of a significant association between the APGAR score and the level of motor development at 3 months is accepted (χ^2^ = 12.963; df = 6; *p* = 0.044).

Although the percentage of premature infants with an APGAR score of 10 and who at 6 months have a level of development similar to that at 5 months is much higher (83.3%) than in the case of premature infants with an APGAR score of 8 (42.9%) and of those with an APGAR score of 9 (55.6%), the difference is not statistically significant (χ^2^ = 3.678; df = 4; *p* = 0.451). Therefore, we can appreciate that the level of the APGAR score is not significantly associated with the level of motor development at 6 months (Table 4).

No significantly different weights of the level of motor development at 9 months were observed according to the APGAR score obtained at birth. The difference is not statistically significant (χ^2^ = 6.114; df = 4; *p* = 0.191). Therefore, we can appreciate that the level of the APGAR score is not significantly associated with the level of motor development at 9 months (Table 4).

The type of muscle tone did not influence the level of motor development at 3 months. The difference is not statistically significant (χ^2^ = 5.350; df = 3; *p* = 0.148). Therefore, we can appreciate that the level of muscle tone score is not significantly associated with the level of motor development at 3 months (Table 5).

If, in the case of the hypotonic form, most premature babies have a motor development equivalent to the age of 4, respectively, 5 months, in the case of the spastic form, there is a high percentage (27.8%) of children who, at 6 months, still have a motor development equivalent to the age of 3 months.

However, a statistically significant association was found (χ^2^ = 7.595; df = 2; *p* = 0.022) between the type of muscle tone and the level of motor development at 6 months (Table 5).

The form of muscle tone is not associated with the level of motor development at 9 months (χ^2^ = 1.044; df = 2; *p* = 0.593) (Table 5).

The bivariate chi-square test (χ^2^) indicated the presence of a significant association between the child’s age at the initial assessment and the level of motor development at 3 months (χ^2^ = 18.731; df = 6; *p* = 0.005). Thus, it was observed that a significant proportion of those who were evaluated for the first time at 3 months (44.4%) have a level of motor development equivalent to the age of 0 months, compared to premature babies whose first evaluation was performed at a younger age and who have a higher motor development at 3 months (Table 6).

Analyzing the level of motor development at 6 months, we notice that those initially assessed at 3 months have a higher proportion of a reduced level of motor development compared to those assessed at younger ages and who at 6 months have reached a higher level of motor development (Table 6). The result of the statistical analysis (χ^2^ = 10.809; df = 4; *p* = 0.029) also shows us that the level of motor development at 6 months differs significantly statistically depending on the age of the child at the initial assessment (Table 6).

There was no significant association between the child’s age at the initial assessment and the level of motor development at 9 months (χ^2^ = 2.343; df = 4; *p* = 0.673) (Table 6).

The results of the statistical analysis showed that premature babies with a low level of motor development at 3 months had a significantly lower birth weight than those who, at 3 months, had a higher weight, as can be seen in the following table (Table 7).

At the 6-month assessment, there are no longer any significant differences in terms of birth weight between children with different levels of motor development. Thus, those with a motor development equivalent to the age of 3 months had an average birth weight of 1768.33 g; those with a motor development equivalent to the age of 4 months had an average birth weight of 1864.55 g; and those with a motor development equivalent to the age of 5 months had an average birth weight of 1956.32 g (Table 7).

As in the case of the 3-month assessment, there is a statistically significant relationship between birth weight and the level of motor development at 9 months, with the mean level of birth weight being significantly higher for those who reached age-matched motor development than those who have it, compared to those who have reached lower levels of motor development (Table 7).

Although in the association analysis between the age at the time of the initial evaluation and the level of motor development, statistically significant relationships were found between the age at the initial time and the motor development at 3 months, respectively, at 6 months, when an evaluation of the achievement of the motor ideal was carried out (expressed as the average level of motor development), it was observed that this achievement of the motor ideal does not differ statistically significantly in any of the evaluations according to the age of the premature at the initial evaluation, as can be seen in the table below (Table 8).

**Figure 5:**
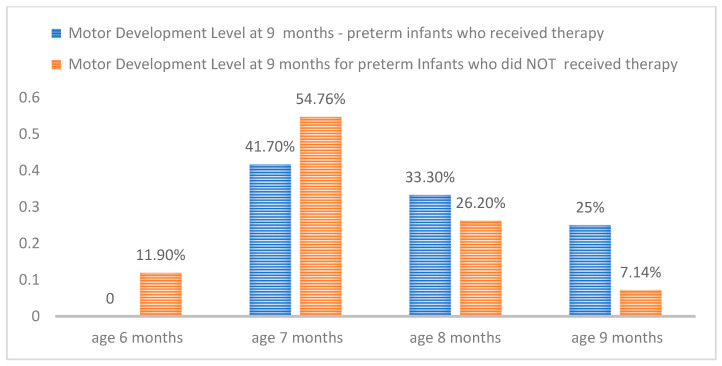
Comparison between the motor development level of the two groups.

In Figure 5, the obtained data regarding the neuromotor development level at the age of 9 months are presented both percentage-wise and comparatively for preterm infants who underwent therapy and the control group. It is evident that preterm infants who did not receive physical therapy and were included in the control group exhibited a larger motor developmental gap compared to those in the therapy group (at 9 months, only 7.14% demonstrated development in accordance with their age).

Figure 5 illustrates the comparison of the level of neuromotor development at 9 months of age between the treatment group and the control group.

## 4. Discussion

The neuromotor development curve of premature infants, in an effort to reach ideal standards, revealed a continuous trajectory with a pronounced increase between the sixth and ninth months. However, significant disparities in motor development were notable, especially in the first 3–6 months of life, when the initiation of Bobath therapy occurred at 3 months of age.

In a cross-sectional study [20] conducted on 308 subjects, comparing the motor performance of preterm infants with that of full-term infants, it was found that preterm infants presented lower motor development scores compared to the normative sample in the age segment between 1 and 12 months. The motor development curve showed a continuous increase in the number of motor skills in premature infants during the first 12 months of life. However, the mean motor acquisition of preterm infants showed a non-linear pattern with a standard indicator of stabilization between 8 and 10 months, leading the researchers to conclude that this category requires tailored follow-up and intervention programs. The study shows that premature infants who started therapy at a younger age, between 1 and 2 months, showed a more favorable motor progression, showing a smaller discrepancy between the initially recorded motor level and the ideal development standards, unlike infants who started therapy at 3 months of age, where the gap was more evident. However, it was highlighted that the time of the first assessment/beginning of the recovery program (at one, two, or three months) does not exert a significant impact on the average achievement of the motor goal established for the age of 9 months (Table 8), with only 25% of infants reaching the ideal motor level of uprighting. This standardization of the degree of development can be attributed, in part, to the benefits brought by the Bobath therapy treatment. As for the rest of the participants, representing 75% of the total, there was a gap of one or two months between the development level and the ideal one. This discrepancy may be related to the fact that age correction was not performed according to the week of birth of the infants. However, even if an age correction were to be implemented, there is a specific delay in motor development in 41% of cases, among the analyzed subjects. Also, Ko J. et al. [21], analyzing motor development between preterm and full-term infants, in a study of 252 subjects between 4 and 9 months of age, identified significant differences in motor development. The motor landmarks evaluated were in four distinct positions (ventral recumbency, supine recumbency, sitting, and standing positions). 

Comparing the level of neuro-motor development at the age of 9 months (Figure 2) between the control group and the one that benefited from therapy, it was noted that the level of motor development of the group that did not benefit from therapy was lower. Thus, among the premature infants included in the control group, there were cases where infants exhibited a developmental level equivalent to that of a 6-month-old (11.90%) at the age of 9 months, and only 7.14% of them at this age initiated verticalization. This discrepancy in neuro-motor development between the two groups is one that guides towards the idea of the need for an early therapeutic intervention.

Dumuids-Vernet et al. [22], in a study carried out in 2022 on the effects of early interventions on locomotor development in infants at risk of motor delay, also concluded that early intervention is essential, determining the most effective results.

In the infant stage, the human brain shows significant plasticity [23], characterized by an active proliferation of dendrites and synapse formation. Experience exerts significant influences on the patterning and structure of the brain, generating structural changes [24], including adjusting the number of synapses developed, regulating their position and functioning, and eliminating redundant synapses. Early intervention can have a marked influence on motor skills, as the motor pathways responsible for the formation of corticospinal tracts show a maturation of myelination as the infant ages [25], and myelination can be activity-dependent [24].

The study showed that gestational age constituted a variable with an unfavorable influence on the progress of neuro-motor development up to the age of 9 months. Also, low birth weight represented a factor that influenced the achievement of the ideal level of motor development, with infants with a lower birth weight registering, on average, a lower level compared to those with a higher birth weight.

Instead, the gender of the infant, the Apgar score, and the muscle tone influenced the neuro-motor development only up to the age of 3 months.

Regarding the influence of factors on the development of gross motor skills in children, from birth to the acquisition of the ability to walk independently, in a review of the specialized literature [26], it was found, as it appears from the present study, that age and weight at birth constitute fundamental biological factors that affect this development.

Children born late prematurely show a more favorable motor development trajectory in contrast to children born extremely prematurely and those born moderately prematurely [27], concluded the researcher Zdzienicka-Chyła et al. in a study carried out in 2018.

This aspect was also highlighted by Maya Yaari et al. in a study that included a number of 149 preterm infants, in which it was emphasized that gestational age represents a factor with a longitudinal effect on the development of fine motor, gross motor, and neonatal neurobehavior. Researchers have also identified the fact that male gender is a predisposing factor of developmental delay [28].

From the statistical interpretation, it was revealed that the gender of the infant exerts an influence on development only up to the age of 3 months. After this period, no significant difference between the two genders was revealed.

Premature birth is not a factor of substantial delay in motor development, concluded Gajewska E. and others in a study in which 419 children (236 boys and 183 girls) were evaluated neurologically and kinetotherapeutically, of which 129 were born prematurely [26]. A combination of several risk factors affected motor development, among which the Apgar score had a significant impact.

Looking at the effectiveness of therapeutic interventions started in the first year of life on the infant’s locomotor development, it was highlighted that interventions started in the early stages, with standardized training protocols that stimulate active movement and frequent and focused training sessions, seem to show remarkable effectiveness in facilitating gross motor and/or locomotor development [22]. Beside therapy, Bobath in the review analyzed the effectiveness of other types of physiotherapy using active and passive components (treadmill walking, time spent on the stomach) [29,30].

Physiotherapy is often necessary for most premature babies, and the main reason is psychomotor retardation, derived from biological immaturity. Among the physiotherapy methods frequently used in the case of premature babies is the Bobath method [31].

Considering the fact that studies [32] have shown that premature babies are accompanied by fine and gross motor impairments until adolescence, it is essential to implement evaluation, monitoring, and early intervention services aimed at this population [33,34,35].

Studies have shown that therapy based on the Bobath concept improves gross motor function [16], postural balance [36,37,38], and coordination, in terms of feeding and swallowing activity [39,40].

The Bobath concept represents one of the most widely used approaches in the field of neurorehabilitation globally, focusing on motor recovery instead of compensation. Although it is widely used, there is confusion at the international level regarding the definition and application of this concept, thus generating difficulties in the interpretation of research results and the evaluation of its effectiveness [41,42].

Other studies [43] used Vojta therapy as an intervention method, one of them stating that the procedure represents a safe kinetotherapeutic approach for premature newborns but requiring further investigations regarding the positive effects.

It is important to emphasize that this investigation represents a pilot study, and the statements regarding the neuro-motor evolution of premature infants and the benefits of Bobath therapy intervention derive from a small sample size. A larger and more diverse sample extension would give a more complex representation of the population and increase the generalizability of the results.

In addition, the limited duration of the study does not fully cover the motor evolution of premature infants, thus imposing longitudinal monitoring, for a comprehensive understanding of the impact and the need for early intervention with the therapy.

Despite these considerations, our study offers the opportunity to introspect on the phenomenon of prematurity and the particular way of neuro-motor development of this population category, requiring exhaustive future studies to complement the previously mentioned limitations.

## 5. Conclusions

Early physiotherapy interventions can have a positive influence on reducing differences in motor development between preterm and full-term infants, thus contributing to their motor progress meeting the ideal standards of development. The analysis showed that there is a difference in the neuro-motor development trajectories of these infants, and the implementation of therapy in the first months of life can have a beneficial effect.

The study identified several factors that influenced the motor development of premature infants. Among these, the most prominent biological factors were gestational age and birth weight.

In order to draw a solid conclusion, however, it is necessary to design future studies that follow this population long-term, to investigate the stimulus-response relationship of therapeutic interventions, to compare the effectiveness of different therapeutic modalities or variations in intervention protocols, and to integrate advanced technologies.

## Figures and Tables

**Table 1:** The main characteristics of the studied sample.

		Frequency	Percent
Gender	Male	22	61.1
	Female	14	38.9
Age at birth (weeks)	34	17	47.2
	35	11	30.6
	36	8	22.2
APGAR score	8	21	58.3
	9	9	25.0
	10	6	16.7
Muscle tone	Hypotonic form	18	50.0
	Spastic form	18	50.0
Age (months): initial assessment	1	7	19.4
	2	11	30.6
	3	18	50.0
Neuromotor development level: initial assessment of the infant	0	19	52.8
	1	9	25.0
	2	4	11.1
	3	4	11.1
Neuromotor development level: assessment of the infant at age 3 months	0	8	22.2
	1	11	30.6
	2	13	36.1
	3	4	11.1
Neuromotor development level: assessment of the infant at age 6 months	3	6	16.7
	4	11	30.6
	5	19	52.8
Neuromotor development level: assessment of the infant at age 9 months	7	15	41.7
	8	12	33.3
	9	9	25.0

**Table 2:** The level of motor development at 3–6–9 months depending on the gender of the infants.

Variable		Level of Development–Evaluation at Infants Age of 3 Months					
		0	1	2	3	Total	*p*
Female	Frequency %	0 0.0%	6 42.9%	8 57.1%	0 0.0%	14 100.0%	0.009
Male	Frequency %	8 36.4%	5 22.7%	5 22.7%	4 18.2%	22 100.0%	
Total	Frequency %	8 22.2%	11 30.6%	13 36.1%	4 11.1%	36 100.0%	
		Level of development–evaluation at infants age of 6 months					
		3	4	5		Total	
Female	Frequency %	0 0.0%	6 42.9%	8 57.1%		14 100.0%	0.081
Male	Frequency %	6 27.3%	5 22.7%	11 50.0%		22 100.0%	
Total	Frequency %	6 16.7%	11 30.6%	19 52.8%		36 100.0%	
		Level of development–evaluation at infants age of 9 months					
		7	8	9		Total	
Female	Frequency %	5 35.7%	5 35.7%	4 28.6%		14 100.0%	0.839
Male	Frequency %	10 45.5%	7 31.8%	5 22.7%		22 100.0%	
Total	Frequency %	15 41.7%	12 33.3%	9 25.0%		36 100.0%	

**Table 3:** Association between age at birth and level of development.

Age at Birth (Weeks)		Developmental Level–Assessment at Infants Age of 3 Months					
		0	1	2	3	Total	*p*
34	Frequency %	5 29.4%	6 35.3%	2 11.8%	4 23.5%	17 100.0%	0.001
35	Frequency %	3 27.3%	5 45.5%	3 27.3%	0 0.0%	11 100.0%	
36	Frequency %	0 0.0%	0 0.0%	8 100.0%	0 0.0%	8 100.0%	
Total	Frequency %	8 22.2%	11 30.6%	13 36.1%	4 11.1%	36 100.0%	
		Developmental level–assessment at infants age of 6 months					
		4	5	6		Total	
34	Frequency %	3 17.6%	7 41.2%	7 41.2%		17 100.0%	0.040
35	Frequency %	3 27.3%	4 36.4%	4 36.4%		11 100.0%	
36	Frequency %	0 0.0%	0 0.0%	8 100.0%		8 100.0%	
Total	Frequency %	6 16.7%	11 30.6%	19 52.8%		36 100.0%	
		Developmental level–assessment at infants age of 9 months					
		7	8	9		Total	
34	Frequency %	12 70.6%	2 11.8%	3 17.6%		17 100.0%	*0.010*
35	Frequency %	2 18.2%	9 81.8%	0 0.0%		11 100.0%	
36	Frequency %	1 12.5%	1 12.5%	6 75.0%		8 100.0%	
Total	Frequency %	15 41.7%	12 33.3%	9 25.0%		36 100.0%	

**Table 4:** Association between APGAR score and developmental level.

APGAR Score		Developmental Level–Assessment at Infants Age of 3 Months					
		0	1	2	3	Total	*p*
8	Frequency %	6 28.6%	8 38.1%	3 14.3%	4 19.0%	21 100.0%	0.440
9	Frequency %	2 22.2%	2 22.2%	5 55.6%	0 0.0%	9 100.0%	
10	Frequency %	0 0.0%	1 16.7%	5 83.3%	0 0.0%	6 100.0%	
Total	Frequency %	8 22.2%	11 30.6%	13 36.1%	4 11.1%	36 100.0%	
		Developmental level–assessment at infants age of 6 months					
		3	4	5		Total	
8	Frequency %	4 19.0%	8 38.1%	9 42.9%		21 100.0%	0.451
9	Frequency %	2 22.2%	2 22.2%	5 55.6%		9 100.0%	
10	Frequency %	0 0.0%	1 16.7%	5 83.3%		6 100.0%	
Total	Frequency %	6 16.7%	11 30.6%	19 52.8%		36 100.0%	
		Developmental level–assessment at infants age of 9 months					
		7	8	9		Total	
8	Frequency %	12 57.1%	6 28.6%	3 14.3%		21 100.0%	*0.191*
9	Frequency %	2 22.2%	4 44.4%	3 33.3%		9 100.0%	
10	Frequency %	1 16.7%	2 33.3%	3 50.0%		6 100.0%	
Total	Frequency %	15 41.7%	12 33.3%	9 25.0%		36 100.0%	

**Table 5:** The association between muscle tone and the level of development.

Muscle Tone		Developmental Level–Assessment at Infants Age of 3 Months					
		0	1	2	3	Total	*p*
Hypotonic form	Frequency %	2 11.1%	8 44.4%	7 38.9%	1 5.6%	18 100.0%	0.148
Spastic form	Frequency %	6 33.3%	3 16.7%	6 33.3%	3 16.7%	18 100.0%	
Total	Frequency %	8 22.2%	11 30.6%	13 36.1%	4 11.1%	36 100.0%	
		Developmental level–assessment at infants age of 6 months					
		3	4	5		Total	
Hypotonic form	Frequency %	1 5.6%	9 50.0%	8 44.4%		18 100.0%	0.022
Spastic form	Frequency %	5 27.8%	2 11.1%	11 61.1%		18 100.0%	
Total	Frequency %	6 16.7%	11 30.6%	19 52.8%		36 100.0%	
		Developmental level–assessment at infants age of 9 months					
		7	8	9		Total	
Hypotonic form	Frequency %	9 50.0%	5 27.8%	4 22.2%		18 100.0%	0.593
Spastic form	Frequency %	6 33.3%	7 38.9%	5 27.8%		18 100.0%	
Total	Frequency %	15 41.7%	12 33.3%	9 25.0%		36 100.0%	

**Table 6:** Association between age at initial assessment and developmental level.

Age–Initial Assessment		Developmental Level–Assessment at Infants Age of 3 Months					
		0	1	2	3	Total	*p*
1	Frequency %	0 0.0%	4 57.1%	3 42.9%	0 0.0%	7 100.0%	0.005
2	Frequency %	0 0.0%	5 45.5%	6 54.5%	0 0.0%	11 100.0%	
3	Frequency %	8 44.4%	2 11.1%	4 22.2%	4 22.2%	18 100.0%	
Total	Frequency %	8 22.2%	11 30.6%	13 36.1%	4 11.1%	36 100.0%	
		Developmental level–assessment at infants age of 6 months					
		3	4	5		Total	
1	Frequency %	0 0.0%	4 57.1%	3 42.9%		7 100.0%	0.029
2	Frequency %	0 0.0%	5 45.5%	6 54.5%		11 100.0%	
3	Frequency %	6 33.3%	2 11.1%	10 55.6%		18 100.0%	
Total	Frequency %	6 16.7%	11 30.6%	19 52.8%		36 100.0%	
		Developmental level–assessment at infants age of 9 months					
		7	8	9		Total	
1	Frequency %	4 57.1%	1 14.3%	2 28.6%		7 100.0%	0.673
2	Frequency %	3 27.3%	5 45.5%	3 27.3%		11 100.0%	
3	Frequency %	8 44.4%	6 33.3%	4 22.2%		18 100.0%	
Total	Frequency %	15 41.7%	12 33.3%	9 25.0%		36 100.0%	

**Table 7:** Association between birth weight and level of development at 3–6–9 months.

Level of Development at Infants Age of 3 Months/Weight (g)				
	N	Mediate	Standard Deviation	*p*
0	8	1738.75	150,849	0.002
1	11	1851.82	175,375	
2	13	2053.08	196,909	
3	4	1830.00	55,976	
Development level at infants age of 6 months/Weight (g)				
3	6	1768.33	164,367	0.125
4	11	1864.55	163,668	
5	19	1956.32	225,862	
Development level at infants age of 9 months/Weight (g)				
7	15	1802.67	126,517	0.029
8	12	1917.50	253,812	
9	9	2026.67	185,876	
Total	36	1896.94	206,948	

**Table 8:** Results of the ANOVA test comparing the level of motor development at 3, 6, and 9 months, respectively, according to the age at the initial assessment.

Age at Assessment Initial		N	Mediate	Standard Deviation	*p*
Developmental level: evaluation at infants age of 3 months	1	7	1.43	0.535	0.678
	2	11	1.55	0.522	
	3	18	1.22	1.263	
	Total	36	1.36	0.961	
Developmental level: evaluation at infants age of 6 months	1	7	4.43	0.535	0.536
	2	11	4.55	0.522	
	3	18	4.22	0.943	
	Total	36	4.36	0.762	
Developmental level: evaluation at infants age of 9 months	1	7	7.71	0.951	0.716
	2	11	8.00	0.775	
	3	18	7.78	0.808	
	Total	36	7.83	0.811	

## Data Availability

Data available on request due to restrictions.
